# *Serratia liquefaciens* KM4 Improves Salt Stress Tolerance in Maize by Regulating Redox Potential, Ion Homeostasis, Leaf Gas Exchange and Stress-Related Gene Expression

**DOI:** 10.3390/ijms19113310

**Published:** 2018-10-24

**Authors:** Mohamed A. El-Esawi, Ibrahim A. Alaraidh, Abdulaziz A. Alsahli, Saud M. Alzahrani, Hayssam M. Ali, Aisha A. Alayafi, Margaret Ahmad

**Affiliations:** 1 Botany Department, Faculty of Science, Tanta University, Tanta 31527, Egypt; 2 UMR CNRS 8256 (B2A), IBPS, Université Paris VI, 75005 Paris, France; margaret.ahmad@upmc.fr; 3 Botany and Microbiology Department, College of Science, King Saud University, P.O. Box 2455, Riyadh 11451, Saudi Arabia; ialaraidh@ksu.edu.sa (I.A.A.); aalshenaifi@ksu.edu.sa (A.A.A.); 434108201@student.ksu.edu.sa (S.M.A.); hayhassan@ksu.edu.sa (H.M.A.); 4 Timber Trees Research Department, Sabahia Horticulture Research Station, Horticulture Research Institute, Agriculture Research Center, Alexandria 21526, Egypt; 5 Biological Sciences Department, Faculty of Science, University of Jeddah, Jeddah 21577, Saudi Arabia; aal_shareaf@hotmail.com; 6 Department of Biology, Xavier University, Cincinnati, OH 45207, USA

**Keywords:** *Serratia liquefaciens* KM4, maize, salt tolerance, antioxidants, gene expression

## Abstract

High salinity mitigates crop productivity and quality. Plant growth-promoting soil rhizobacteria (PGPR) improve plant growth and abiotic stress tolerance via mediating various physiological and molecular mechanisms. This study investigated the effects of the PGPR strain *Serratia liquefaciens* KM4 on the growth and physiological and molecular responsiveness of maize (*Zea mays* L.) plants under salinity stress (0, 80, and 160 mM NaCl). High salinity significantly reduced plant growth and biomass production, nutrient uptake, leaf relative water content, pigment content, leaf gas exchange attributes, and total flavonoid and phenolic contents in maize. However, osmolyte content (e.g., soluble proteins, proline, and free amino acids), oxidative stress markers, and enzymatic and non-enzymatic antioxidants levels were increased in maize under high salinity. On the other hand, *Serratia liquefaciens* KM4 inoculation significantly reduced oxidative stress markers, but increased the maize growth and biomass production along with better leaf gas exchange, osmoregulation, antioxidant defense systems, and nutrient uptake under salt stress. Moreover, it was found that all these improvements were accompanied with the upregulation of stress-related genes (*APX*, *CAT*, *SOD*, *RBCS*, *RBCL*, *H^+^-PPase*, *HKT1*, and *NHX1*), and downregulation of the key gene in ABA biosynthesis (*NCED*). Taken together, the results demonstrate the beneficial role of *Serratia liquefaciens* KM4 in improving plant growth and salt stress tolerance in maize by regulating ion homeostasis, redox potential, leaf gas exchange, and stress-related genes expression.

## 1. Introduction

Soil salinity stress affects crops growth and performance, and represents a main threat to the sustainable agricultural development worldwide [1,2,3]. It severely affects the physiological processes in plants, including lipid metabolism, protein synthesis, ion homeostasis, photosynthesis, and nitrogen fixation [3,4]. It minimizes water uptake by roots and causes over-production of toxic ions [5], resulting in the generation of toxic free radicals that cause oxidative damage [6]. At high salt concentrations, the excess in Na^+^ content and ethylene reduces the uptake of the macronutrients and negatively affects plant function [5,7,8]. High salinity also represses plant cell division and elongation which ultimately affects the root growth [9,10]. To mitigate the oxidative damage and negative impacts of salt stress, crops have developed various self-defense mechanisms such as compartmentalization of ions, production of compatible osmolytes, regulation of photosynthetic pathways, induction of phytohormones, and upregulation of antioxidants such as catalase (CAT), ascorbic acid (AsA), glutathione reductase (GR), proline, peroxidase (POD), and superoxide dismutase (SOD) [6,11,12,13,14].

Several approaches have been undertaken to physiologically and genetically characterize plants and even other organisms and consequently improve their growth and performance [15,16,17,18,19,20,21,22,23,24,25,26,27,28,29,30,31]. Utilization of plant growth promoting rhizobacteria (PGPR) represents one of those efficient approaches and showed great potential in enhancing plant growth and performance by providing nitrogen and phosphorous nutrition, phosphate solubilization, production of phytohormones, and control of pathogens [2,9]. Earlier reports have also revealed the efficiency of PGPR in ameliorating the negative impacts of salinity via mediating different physiological and molecular mechanisms [9,32,33]. Such mechanisms involve the activation of antioxidant systems, synthesis of phytohormones, altering root system, and synthesis of osmolytes, including proline, soluble proteins, and sugars [9,32,33]. For example, El-Esawi et al. [32] reported that *Bacillus firmus* SW5 improved soybean growth and salinity stress tolerance in soybean via modulating root architecture, antioxidants, and stress-related gene expression. Additionally, PGPR generate 1-aminocyclopropane-1-carboxylate (ACC) deaminase enzyme which mitigates salinity-induced stress ethylene and consequently enhances plant growth [34]. Furthermore, PGPR played a crucial role in alleviating the oxidative damage and negative impacts of other environmental factors, including water deficit, drought, low and high temperature, and heavy metal stress [35]. 

Maize (*Zea mays* L.) is one of the major cereal crops cultivated worldwide for its crucial use in food and industry. Maize is a moderately salt-sensitive crop subjected to salt toxicity under irrigation which influences plant growth and performance [3,8]. Thus, further research should be conducted to mitigate the negative impacts of salinity on maize growth and performance. Considering the effective role of PGPR in ameliorating high salinity and maintaining plant growth, the present investigation aimed at studying the role of *Serratia liquefaciens* KM4, for the first time, in ameliorating the adverse impacts of high salinity on the growth and physiological traits of maize plants. Moreover, this study investigated the influence of *Serratia liquefaciens* KM4 on osmolytes accumulation, antioxidant enzymes activities, and expression of genes mediating salt tolerance in salt-treated maize plants.

## 2. Results 

### 2.1. Plant Growth and Biomass Yield

*Serratia liquefaciens* KM4 grew up to 300 mM NaCl in the nutrient broth medium. Effect of *Serratia liquefaciens* KM4 on the growth and biomass of maize subjected to salt stress was studied (Table 1). Data presented in Table 1 show significant reductions in the root and shoot lengths as well as fresh and dry weights of maize plants under salinity levels (80 and 100 mM NaCl) as compared with control maize plants. Maximum decrease in maize growth and biomass was recorded at the high salt concentration (160 mM NaCl) as compared with control plants. However, under salinity stress, *Serratia liquefaciens* KM4-inoculated maize plants exhibited better growth and biomass yield as compared with non-inoculated maize plants (Table 1). Moreover, under control conditions, inoculated maize plants exhibited better growth and biomass production as compared with non-inoculated maize plants (Table 1).

### 2.2. Mineral Uptake, Pigment Contents, Leaf Relative Water Content (LRWC), and Antioxidant Capacity

Maize plants exhibited increased contents of Na^+^ and Cl^−^ ions and reduced levels of K^+^ ad Ca^2+^ ions under salinity stress levels (80 and 160 mM NaCl) as compared with control plants (Table 2). However, under saline conditions, *Serratia liquefaciens* KM4 inoculation significantly mitigated the uptake of Na^+^ and Cl^−^ ions but enhanced the accumulation of K^+^ ad Ca^2+^ ions inside maize cells, as compared with non-inoculated maize plants (Table 2). Moreover, under control conditions, inoculated maize plants exhibited significant reductions in Na^+^ and Cl^−^ contents as well as improvements in K^+^ and Ca^2+^ contents as compared with non-inoculated maize plants (Table 2). 

Results presented in Table 3 show that the increase in NaCl concentration was accompanied with significant decreases in leaf relative water content (LRWC) and pigment content (chlorophyll and carotenoids) in maize leaves as compared with control plants. The maximum decreases in LRWC and pigment content were noted at the high saline concentration (160 mM NaCl) as compared with control plants. However, under salinity stress, *Serratia liquefaciens* KM4-inoculated maize plants exhibited significant increases in LRWC and pigment content as compared with non-inoculated maize plants (Table 3). Moreover, under control conditions, inoculated maize plants exhibited better LRWC and pigment content as compared with non-inoculated maize plants (Table 3).

Under salinity stress, *Serratia liquefaciens* KM4-inoculated maize plants exhibited significantly reduced IC_50_ values for DPPH assay and higher antioxidant activities as compared with non-inoculated maize plants (Table 3). Moreover, under control conditions, inoculated maize plants exhibited significantly reduced IC_50_ values and higher antioxidant activities as compared with non-inoculated maize plants (Table 3).

### 2.3. Levels of Proline, Soluble Sugars, Soluble Proteins, Total Free Amino Acids, Phenols, and Flavonoids

Soil salinity significantly influenced the levels of osmoprotectants and antioxidants in maize leaves (Table 4). Data presented in Table 4 show significant reductions in the contents of soluble sugars, phenols, and flavonoids in maize plants under salinity levels (80 and 100 mM NaCl) as compared with control maize plants, and the maximum decrease was noted at the high salt concentration (160 mM NaCl). However, significant increases in the contents of proline, soluble protein and total free amino acid were recorded in maize plants subjected to salinity stress, and the maximum increase was noted at the high salt concentration (160 mM NaCl) as compared with control plants (Table 4).

On the other hand, under salinity stress, *Serratia liquefaciens* KM4-inoculated maize plants exhibited significantly increased levels of proline, soluble sugars, soluble protein, total free amino acid, phenols, and flavonoids as compared with non-inoculated maize plants (Table 4). Moreover, under control conditions, inoculated maize plants exhibited significantly enhanced osmoprotectants and antioxidants contents as compared with non-inoculated maize plants (Table 4).

### 2.4. Estimation of Oxidative Stress Markers

Soil salinity significantly influenced the levels of oxidative stress markers in maize leaves (Table 5). Table 5 shows a significant increase in the levels of hydrogen peroxide (H_2_O_2_), malondialdehyde (MDA), and electrolyte leakage (EL) in maize plants under salinity levels (80 and 100 mM NaCl) as compared with control maize plants, and the maximum increase was noted at the high salt concentration (160 mM NaCl). On the other hand, under salinity stress, *Serratia liquefaciens* KM4-inoculated maize plants exhibited significantly reduced levels of H_2_O_2_, MDA, and EL as compared with non-inoculated maize plants (Table 5). Moreover, under control conditions, inoculated maize plants exhibited significantly reduced oxidative stress markers levels as compared with non-inoculated maize plants (Table 5).

### 2.5. Measurement of Gas-Exchange Parameters

Levels of transpiration rate (*E*), net photosynthesis rate (*P_n_*), and stomatal conductance (*g_s_*) were significantly decreased in maize plants subjected to salinity stress (80 and 100 mM NaCl) as compared with control maize plants, and the maximum decrease was recorded at the high salt concentration (160 mM NaCl) (Table 5). On the other hand, under salinity stress, *Serratia liquefaciens* KM4-inoculated maize plants exhibited higher levels of gas-exchange parameters as compared with non-inoculated maize plants (Table 5). Moreover, under control conditions, inoculated maize plants exhibited significantly increased levels of gas-exchange attributes as compared with non-inoculated maize plants (Table 5).

### 2.6. Activities of Antioxidant Enzymes and Levels of Non-Enzymatic Antioxidants

**Figure 1:**
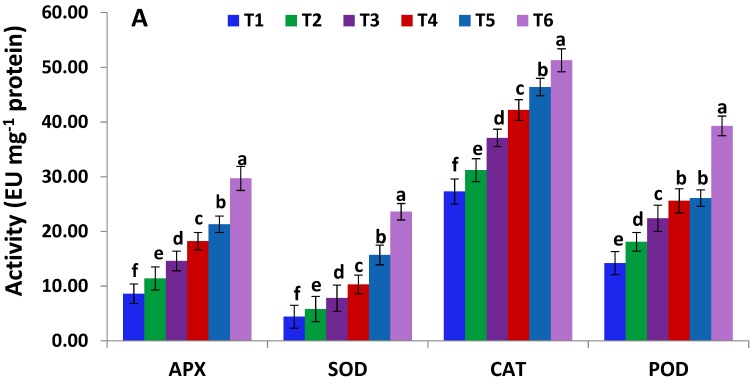

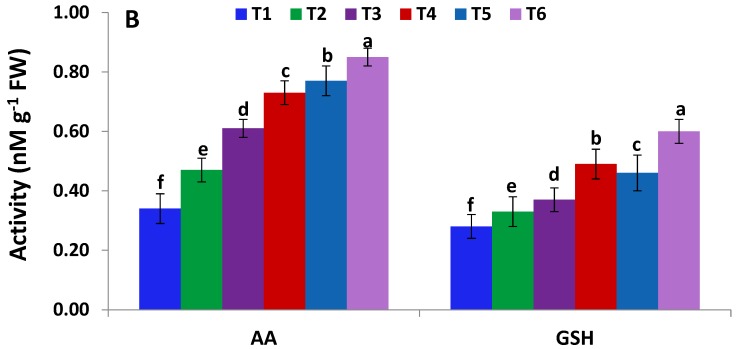
Levels of enzymatic (**A**) and non-enzymatic (**B**) antioxidants in maize plants in the absence and presence of *Serratia liquefaciens* KM4 under different saline concentrations. Data are means ± SE (*n* = 5). Different letters indicate significant differences among treatments. T1, non-inoculated control plants; T2, *Serratia liquefaciens* KM4-inoculated plants; T3, 80 mM NaCl-treated plants; T4, plants primed with 80 mM NaCl and *Serratia liquefaciens* KM4; T5, 160 mM NaCl-treated plants; T6, plants primed with 160 mM NaCl and *Serratia liquefaciens* KM4.

The effects of *Serratia liquefaciens* KM4 on the enzymatic and non-enzymatic antioxidants levels in maize plants under salinity stress were investigated (Figure 1). The activities of antioxidant enzymes (APX, SOD, CAT, and POD) and levels of non-enzymatic antioxidants (AA and GSH) were significantly increased in maize plants subjected to salinity stress (80 and 100 mM NaCl) as compared with control plants, and the maximum increase was recorded at the high salt concentration (160 mM NaCl) (Figure 1). On the other hand, under salinity stress, *Serratia liquefaciens* KM4-inoculated maize plants exhibited higher levels of the enzymatic and non-enzymatic antioxidants as compared with non-inoculated maize plants (Figure 1). Moreover, under control conditions, inoculated maize plants exhibited significantly increased levels of the enzymatic and non-enzymatic antioxidants as compared with non-inoculated maize plants (Figure 1).

### 2.7. Gene Expression Analysis 

**Figure 2:**
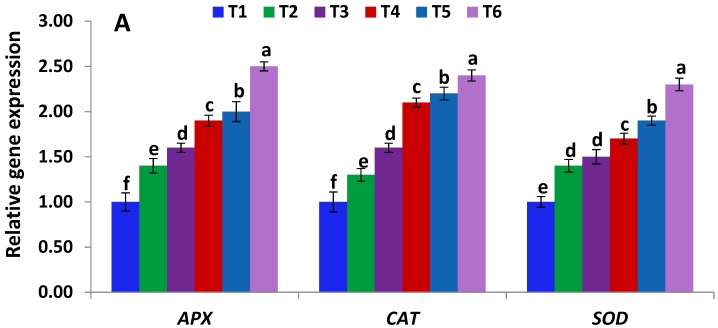

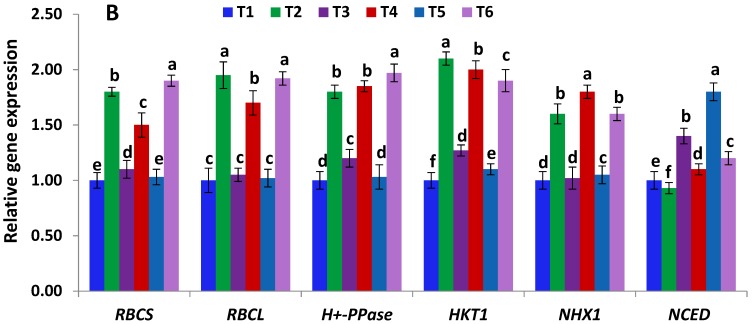
Expression levels of antioxidant genes (**A**) and stress-related genes (**B**) of maize in absence and presence of *Serratia liquefaciens* KM4 under different saline concentrations. Data are means ± SE (*n* = 5). Different letters indicate significant differences among treatments (*p* ≤ 0.05). T1, non-inoculated control plants; T2, *Serratia liquefaciens* KM4-inoculated plants; T3, 80 mM NaCl-treated plants; T4, plants primed with 80 mM NaCl and *Serratia liquefaciens* KM4; T5, 160 mM NaCl-treated plants; T6, plants primed with 160 mM NaCl and *Serratia liquefaciens* KM4.

The effects of *Serratia liquefaciens* KM4 on the expression of genes conferring salt tolerance was investigated. Under salinity stress, *Serratia liquefaciens* KM4-inoculated maize plants exhibited higher expression levels of the antioxidant genes (*APX*, *CAT*, *SOD*), Rubisco and photosynthesis-encoding genes (*RBCS* and *RBCL*), and genes mediating ion balance (*H^+^-PPase*, *HKT1*, *NHX1*), but exhibited lower level of the key gene of ABA synthesis (*NCED*) as compared with non-inoculated maize plants (Figure 2). Moreover, under control conditions, inoculated maize plants exhibited higher expressions levels for all the analyzed genes with the exception of *NCED* gene as compared with non-inoculated maize plants (Figure 2).

## 3. Discussion

Earlier reports revealed the key role of plant-growth promoting rhizobacteria, including *Serratia* spp., in improving plant growth and environmental stress tolerance [36,37,38,39]. However, the current study revealed, for the first time, that the PGPR strain *Serratia liquefaciens* KM4 could enhance maize growth under normal and high salinity conditions. The effects observed are consequence of the PGPRs properties of *Serratia liquefaciens* KM4 strain. *Serratia liquefaciens* KM4 inoculation significantly improved plant growth, biomass yield and acquisition of calcium and potassium under control and saline conditions, supporting the possibility that KM4 improves salt tolerance in maize by promoting plant growth (Table 1 and Table 2). Moreover, *Serratia liquefaciens* KM4 significantly induced the photosynthetic pigment content and the expression of key genes mediating Rubisco and photosynthesis (*RBCS* and *RBCL*) in maize plants (Table 3, Figure 2), demonstrating that *Serratia liquefaciens* KM4 could enhance the efficiency of photosynthesis under high salinity stress. Those results were in concordance with that of Chen et al. [40] who demonstrated that *Bacillus amyloliquefaciens* SQR9 might enhance photosynthesis efficiency by the induction of pigment content and upregulation of *RBCS* and *RBCL* genes in maize subjected to saline conditions. Moreover, *Arabidopsis helleri* inoculated with different bacterial strains exhibited an induction in various proteins related to photosynthesis and abiotic stress [41]. 

Under saline conditions, the toxicity of Na^+^ could be mitigated in plant cells through various mechanisms, including restricting Na^+^ uptake, recycling Na^+^ from the xylem stream to root system, sequestering Na^+^ into vacuoles and exporting it out of cells [42]. Processes of Na^+^ sequestration, export and recirculation are mediated by Na^+^/H^+^ antiporters (*NHX1* and *NHX7*) and ion balance regulators (*H^+^-PPase* and *HKT1*) [40]. In the current study, *Serratia liquefaciens* KM4 inoculation significantly mitigated the contents of Na^+^ and Cl^−^ and upregulated the expression of *NHX1*, *H^+^-PPase* and *HKT1* genes in maize under control and saline conditions (Table 2, Figure 2), suggesting that KM4 decreases the contents of such toxic ions in maize plants by sequestering them into vacuoles, exporting them out of cells and recirculating them from shoot to root, thereby ameliorating ion toxicity and enhancing salinity tolerance. Moreover, *Serratia liquefaciens* KM4 downregulated the expression of *NCED* gene mediating ABA biosynthesis. Our findings are supported by previous reports. For instance, Zhang et al. [43] indicated that *B. subtilis* GB03 inoculation enhanced salinity stress tolerance in *Arabidopsis thaliana* by inducing *HKT1* gene expression in shoots. Schilling et al. [44] revealed that barley plants overexpressing *H^+^-PPase* exhibited high salt tolerance. Na^+^/H^+^ antiporters (*NHX1* and *NHX7*) in *Arabidopsis* sequestered Na^+^ ions into vacuoles and expelled them out of cells [45,46]. Moreover, Chen et al. [40] also reported that *Bacillus amyloliquefaciens* SQR9 might enhance salt tolerance and mitigate ion toxicity in maize by downregulating the expression of *NCED* gene and upregulating the expression levels of *NHX1*, *H^+^-PPase*, and *HKT1* genes. Bharti et al. [47] also revealed that *Dietzia natronolimnaea*-inoculated wheat plants exhibited higher expression levels of important transport proteins which regulate toxic ions compartmentalization.

Under high soil salinity, plants accumulate reactive oxygen species which result in severe oxidative damage [48]. As a result, plants induce the biosynthesis of soluble solutes and upregulate their antioxidants in order to mitigate the high salinity-induced inhibitory effects and maintain cell homeostasis and cell water balance [40]. Inoculating plants with PGPR or rhizobia could enhance the production of soluble sugars, soluble proteins, proline, and choline in plants under high salinity, and consequently tolerate oxidative and osmotic stresses [49,50]. In the current study, high salinity reduced soluble sugars content but induced the biosynthesis of proline and total soluble proteins in maize plants (Table 4). Enhanced soluble protein level under salt stress could be attributed to the induction of stress-related proteins biosynthesis [51]. Such soluble proteins could adjust the osmosis in plant cells. Moreover, cell homeostasis is regulated by soluble sugars [52]. Modulating soluble sugars content under high salinity results in modifications in CO_2_ assimilation, expression of certain genes and enzyme activities [52]. Khan et al. [53] also reported proline accumulation in *Brassica juncea* under high salinity stress. Proline regulates osmoregulation of plant cells, maintains water balance in cells, scavenges ROS, and enhances photosynthesis and nitrogen fixation under saline conditions [54]. On the other hand, *Serratia liquefaciens* KM4 inoculation improved the levels of free amino acids, proline, total soluble proteins and soluble sugars in salinity-stressed maize plants (Table 4). This might induce salinity tolerance in maize by mediating osmosis. Chen et al. [40] also reported high levels of soluble sugars and osmolytes in *Bacillus amyloliquefaciens* SQR9-inoculated maize under saline conditions, thereby enhanced salinity tolerance. Moreover, *Arthrobacter* sp. SU18 and *Bacillus subtilis* SU47 induced the levels of proline and soluble sugars in wheat subjected to high salinity [55].

In the current study, salinity stress decreased LRWC, total phenolic and flavonoid contents, and gas exchange parameters in maize, causing water uptake reduction and root damage (Table 3, Table 4 and Table 5). On the other hand, *Serratia liquefaciens* KM4 induced LRWC, gas exchange parameters, and biosynthetic pathways of phenols and flavonoids in maize subjected to saline conditions, thus improving plant tolerance to salinity stress. Our results are supported by previous reports. For instance, Bahadur et al. [56] reported phenols accumulation in pea plants inoculated with PGPR, which in turn augmented plant tolerance to fungal infection. Furthermore, *Serratia liquefaciens* KM4-inoculated maize plants exhibited significant reductions in lipid peroxidation, hydrogen peroxide production, and electrolyte leakage under salinity stress (Table 5). This might be explained that *Serratia liquefaciens* KM4 regulate the functions of membrane by scavenging ROS over-production. Those findings are in agreement with the results of Han et al. [57] reported in PGPR-inoculated maize and white clover plants.

To scavenge reactive oxygen species and alleviate cellular toxicity, plants also activate their enzymatic and non-enzymatic antioxidants. In the current study, *Serratia liquefaciens* KM4 inoculation significantly enhanced the levels of antioxidant enzymes (APX, CAT, SOD, and POD) and non-enzymatic redox antioxidants (AA and GSH) under saline conditions (Figure 1). These results are supported by the findings of Hashem et al. [58] who recorded induced antioxidants levels in PGPR-inoculated crops. This might help maintaining the photosynthetic electron transport chain, thereby eliminate free radicals [59]. AA and GSH are crucial components of ROS-scavenging pathway. Under salt stress, redox reactions happen in the ascorbate-glutathione cycle in order to alleviate hydrogen peroxide and oxidative damage. Moreover, *Serratia liquefaciens* KM4 inoculation significantly upregulated the genes related to salinity tolerance and the antioxidant enzyme-encoding genes (*APX*, *CAT*, *SOD*) under control and saline conditions (Figure 2), in concordance with the induced antioxidant enzyme activities. The higher increase in enzymatic and non-enzymatic antioxidant levels in *Serratia liquefaciens* KM4-inoculated maize plants indicates that KM4 also induces antioxidant defense systems in maize in order to eliminate toxic free radicals and confer enhanced salt tolerance. 

In conclusion, *Serratia liquefaciens* KM4 exhibited great potential in promoting maize growth, even in the presence of salt by inducing the accumulation of soluble solutes, levels of enzymatic and non-enzymatic antioxidants, photosynthesis efficiency, and expression of stress-related genes, as well as downregulating the expression of genes mediating ABA biosynthesis. 

## 4. Materials and Methods 

### 4.1. Test of Salt Tolerance of Serratia Liquefaciens KM4

The bacterial strain *Serratia liquefaciens* KM4 was isolated from the rhizospheric soil of maize plants grown in Suez Canal region of Egypt and exhibited great potential to solubilize inorganic phosphate and generate siderophores and indole acetic acid [60,61]. Here, the salt tolerance of *Serratia liquefaciens* KM4 was tested in nutrient broth supplemented with 100, 300 and 450 mM NaCl. Growth of bacteria was determined by reading the absorbance at 600 nm after 24, 48, 72 and 96 h of incubation at 29 °C.

### 4.2. Bacterial Inoculation and Plant Growth 

*Serratia liquefaciens* KM4 was grown in nutrient broth at 28 °C for 4 days. Cells were collected following centrifugation at 2000 *g* for 5 min. Pellets were then re-suspended in sterile H_2_O, and bacterial suspension of 10^8^ colony-forming units (CFU) mL^−1^ was used for inoculating maize plants.

Seeds of maize (*Zea mays* L. cv. Giza 2) received from Giza Agriculture Research Center in Egypt were sterilized in NaClO (5%, *v/v*) for 5 min, washed with sterile H_2_O, and left to grow at 24 °C for 4 days. The 4-day old maize seedlings of uniform growth were used and split into 2 groups; one group was treated with a fresh nutrient broth for 20 min and the other group was inoculated with *Serratia liquefaciens* KM4 suspension for 20 min. The seedlings of both groups were transplanted into plastic pots filled with autoclaved soil which consists of sand, peat, and perlite (1:1:1, *v/v/v*). Pots were kept in a randomized block design with three replications in a growth chamber under growth conditions of 16/8 h day/night photoperiod, 25/21 °C day/night temperature, and 75/80% day/night humidity. After transplantation immediately, the plants were irrigated daily with a Hoagland nutrient solution supplied with 0 (control), 80 and 160 mM NaCl through the whole experimental period (24 days). Therefore, the treatments used in the experimental design were as follows: (i) non-inoculated control plants (T1); (ii) *Serratia liquefaciens* KM4-inoculated plants (T2); (iii) 80 mM NaCl-treated plants (T3); (iv) plants primed with 80 mM NaCl and *Serratia liquefaciens* KM4 (T4); (v) 160 mM NaCl-treated plants (T5); and (vi) plants primed with 160 mM NaCl and *Serratia liquefaciens* KM4 (T6). After 24 days of transplantation, maize plants were collected and used for further analyses.

### 4.3. Estimation of Growth and Biomass Yield

The harvested maize plants were cleaned with distilled H_2_O. Lengths and fresh weights (FW) of shoot and root were determined. Dry weights (DW) of shoot and root were also determined following drying at 70 °C for 4 days.

### 4.4. Estimation of Na^+^, Cl^−^, Ca^2+^, and K^+^ Content

Dried leaf powder (0.1 g) was dissolved in 2 mL of 80% perchloric acid and 10 mL of concentrated H_2_SO_4_ and the mixture was diluted with sterile H_2_O to 100 mL. Na^+^, Cl^−^, Ca^2+^ and K^+^ contents were estimated using flame photometry following the methods previously reported by Williams and Twine [62] and Wolf [63].

### 4.5. Determination of Pigment Contents

Contents of chlorophyll and carotenoids in maize leaf were determined as previously described by Lichtenthaler and Wellburn [64]. Optical density was taken at 663, 645, and 453 nm to estimate chlorophyll a, chlorophyll b, and carotenoids, respectively, using 80% acetone as a blank.

### 4.6. Estimation of Osmoprotectant Contents

Leaf proline content was estimated as reported by Bates et al. [65]. Anthrone sulfuric acid method was utilized to determine the total leaf soluble sugar level as reported by Irigoyen et al. [66]. Bradford method was utilized to determine the total leaf soluble protein level as previously reported by Bradford [67]. Total leaf free amino acids level was estimated as described by Lee and Takanashi [68].

### 4.7. Determination of Phenloic and Flavonoid Contents

To extract phenols and flavonoids in fresh leaves, 50 mg fresh tissues were homogenized in 80% ethanol (0.5 mL), centrifuged and supernatants were then pooled. The total phenolic content was estimated following Folin–Ciocalteu assay as previously reported by Zhang et al. [69] using gallic acid as a standard. The total flavonoid content was calculated following aluminum chloride calorimetric method as reported by Chang et al. [70] using quercetin as a standard.

### 4.8. Estimation of Oxidative Stress Markers

To estimate hydrogen peroxide (H_2_O_2_) content in fresh leaves, 50 mg fresh tissues were homogenized in 0.5 mL TCA (0.1%), and then centrifuged. H_2_O_2_ content was calculated as reported by Velikova et al. [71], and results were expressed as µmol g^−1^ FW.

Malondialdehyde (MDA) level in fresh leaves was estimated following the protocol previously reported by Rao and Sresty [72]. MDA content was expressed as nmol/g FW tissue. Electrolyte leakage of leaf was estimated as reported by Dionisio-Sese and Tobita [73].

### 4.9. Determination of Antioxidant Capacity

Antioxidant capacity of maize leaves was measured following 2,2′-diphenypicrylhydrazyl (DPPH) assay reported by Pyrzynska and Pekal [74]. The absorbance was spectrophotometrically examined at 517 nm, and DPPH values were expressed as IC_50_ in mg mL^−1^.

### 4.10. Determination of LRWC and Gas-Exchange Parameters

LRWC was estimated as reported by Garíca-Mata and Lamattina [75]. Transpiration rate (*E*), net photosynthesis rate (*P_n_*) and stomatal conductance (*g_s_*) in maize leaves were estimated early morning using a portable gas-exchange system *LCpro+* (ADC BioScientific Ltd., Hertfordshire, UK) as previously reported by Holá et al. [76].

### 4.11. Assays of Antioxidant Enzyme Activities

Activities of catalase (CAT), ascorbate peroxidase (APX), superoxide dismutase (SOD), and peroxidase (POD) enzymes were estimated in fresh leaves using the methodology of Zhang and Kirkham [77]. Briefly, 0.3 g of fresh tissue was homogenized in 4 mL extraction buffer containing 1% PVP, PBS (50 mM), and EDTA (0.2 mM), and then centrifuged at the highest speed at 4 °C for 8 min. The absorbance was taken at 290 nm (APX) or 240 nm (CAT) or 470 m (POD). SOD activity was also measured in fresh leaves as previously reported by Bradford [67]. Briefly, leaf tissue was homogenized in 0.2 M phosphate buffer, and centrifuged at the highest speed at 4 °C for 8 min. Supernatant absorbance was then read at 560 nm.

### 4.12. Levels of Non-Enzymatic Antioxidants

Ascorbic acid (AA) content in maize leaves was estimated using the protocol reported by Mukherjee and Choudhuri [78]. Absorbance was then determined at 530 nm and ascorbic acid calibration curve was served as a standard. Glutathione (GSH) in leaf was estimated as described by Yu et al. [79] and was expressed as nM g^−1^ FW.

### 4.13. Gene Expression Analysis

Quantitative RT-PCR analysis was performed to investigate the expression of 9 genes conferring salinity tolerance in maize leaf. Those 9 genes include 3 antioxidant genes (CAT, APX, and SOD), *RBCL* (encoding ribulose-1,5-bisphosphate carboxylase/oxygenase large subunit), *RBCS* (encoding ribulose-1,5-bisphosphate carboxylase/oxygenase (Rubisco) small subunit), *H^+^-PPase* (encoding H^+^-pumping pyrophosphatase), *NHX1* (encoding Na^+^/H^+^ antiporter), *HKT1* (encoding high-affinity K^+^ transporter 1), and *NCED* (encoding 9-*cis*-epoxycarotenoid dioxygenase). Total RNA extraction was conducted from fresh leaves of maize using RNeasy Plant Mini kit (Qiagen, Hilden, Germany). RNase-Free DNase Set (Qiagen, Hilden, Germany) was utilized to remove contaminating DNA. Reverse Transcription kit (Qiagen, Hilden, Germany) was then utilized to synthesize cDNA. Quantitative RT-PCR was performed in triplicates following the manufacturer’s protocol of QuantiTect SYBR Green PCR kit (Qiagen, Hilden, Germany). PCR thermal conditions were adjusted as follows: 94 °C for 10 min; 40 cycles of 95 °C for 20 s, 60 °C for 30 s, 72 °C for 2 min and 72 °C for 4 min. Gene specific-primers [12,40] were utilized for amplification. Amplification specificity was verified by melt-curve analysis. *TUB* (encoding α-tubulin) was utilized as an internal reference [40] and the relative expression levels were estimated following 2^−ΔΔ*C*t^ method [80].

### 4.14. Statistical Analysis

Statistical analysis was conducted using one-way analysis of variance (ANOVA) and Duncan’s multiple range test. Values are means ± SE (*n* = 5) and differ significantly at *p* ≤ 0.05.

## Figures and Tables

**Table 1:** Maize growth and biomass production in the presence or absence of *Serratia liquefaciens* KM4 under salt stress.

NaCl (mM)	*Serratia liquefaciens* KM4	Shoot Length (cm)	Shoot Fresh Weight (g·plant^−1^)	Shoot Dry Weight (g·plant^−1^)	Root Length (cm)	Root Fresh Weight (g·plant^−1^)	Root Dry Weight (g·plant^−1^)
0	−KM4	32.4 ± 1.88 b	5.9 ± 0.41 b	0.58 ± 0.12 b	20.5 ± 1.21 b	2.71 ± 0.19 b	0.38 ± 0.16 b
	+KM4	35.7 ± 1.45 a	6.4 ± 0.35 a	0.63 ± 0.14 a	22.9 ± 1.32 a	2.98 ± 0.18 a	0.43 ± 0.15 a
80	−KM4	26.2 ± 1.29 d	5.1 ± 0.38 c	0.52 ±0.23 d	17.6 ± 1.21 d	2.32 ± 0.22 d	0.32 ±0.17 d
	+KM4	30.1 ± 1.17 c	5.4 ± 0.41 b	0.55 ± 0.16 c	19.2 ± 1.17 c	2.59 ± 0.18 c	0.36 ± 0.15 c
160	−KM4	22.4 ± 1.11 f	3.9 ± 0.27 e	0.42 ± 0.18 f	14.3 ± 1.15 f	1.98 ± 0.15 f	0.23 ± 0.12 f
	+KM4	24.3 ± 1.25 e	4.3 ± 0.31 d	0.46 ± 0.15 e	16.6 ± 1.24 e	2.22 ± 0.17 e	0.27 ± 0.11 e

Values indicate means ± SE (*n* = 5). In the same column, values followed by similar letters are not significantly different.

**Table 2:** Mineral uptake in leaves of maize plants in the presence or absence of *Serratia liquefaciens* KM4 under saline stress.

NaCl (mM)	*S. liquefaciens* KM4	Na^+^ (mg g^−1^ DW)	Cl^−^ (mg g^−1^ DW)	Ca^2+^ (mg g^−1^ DW)	K^+^ (mg g^−1^ DW)
0	−KM4	5.3 ± 0.11 e	8.5 ± 0.15 e	17.3 ± 0.13 b	29.4 ± 0.21 b
	+KM4	4.2 ± 0.14 f	7.1 ± 0.12 f	19.4 ± 0.12 a	31.6 ± 0.19 a
80	−KM4	8.9 ± 0.16 c	11.4 ± 0.17 c	12.8± 0.14 d	22.8 ± 0.11 d
	+KM4	6.5 ± 0.12 d	10.0 ± 0.14 d	15.2 ± 0.19 c	26.7 ± 0.18 c
160	−KM4	16.6 ± 0.11 a	14.6 ± 0.12 a	10.1 ± 0.11 e	16.2 ± 0.22 f
	+KM4	12.8 ± 0.14 b	12.1 ± 0.15 b	13.6 ± 0.16 d	18.3 ± 0.24 e

Values indicate means ± SE (*n* = 5). In the same column, values followed by similar letters are not significantly different.

**Table 3:** Leaf relative water content (LRWC), antioxidant activity (DPPH, μg mL^−1^), and contents of chlorophyll (Chl) and carotenoid in maize leaves in the presence or absence of *Serratia liquefaciens* KM4 under saline conditions.

NaCl (mM)	*S. liquefaciens* KM4	LRWC (%)	DPPH (IC_50_)	Chl a (mg g^−1^ FW)	Chl b (mg g^−1^ FW)	Total Chl (mg g^−1^ FW)	Carotenoid (mg g^−1^ FW)
0	−KM4	86.1 ± 1.23 b	0.62 ± 0.07 a	3.31 ± 0.12 b	1.72 ± 0.11 b	5.03 ± 0.11 b	0.29 ± 0.05 e
	+KM4	87.9 ± 2.12 a	0.56 ± 0.05 b	3.74 ± 0.14 a	1.97 ± 0.09 a	5.71 ± 0.17 a	0.34 ± 0.04 a
80	−KM4	78.4 ± 1.87 d	0.51 ± 0.08 c	2.52 ± 0.18 d	1.36 ± 0.08 d	3.88 ± 0.09 e	0.24 ± 0.08 d
	+KM4	82.6 ± 2.41 c	0.44 ± 0.09 d	3.18 ± 0.11 c	1.61 ± 0.06 c	4.79 ± 0.12 c	0.28 ± 0.04 b
160	−KM4	72.3 ± 2.11 f	0.43 ± 0.05 d	2.11 ± 0.17 e	1.11 ± 0.09 e	3.22 ± 0.16 f	0.20 ± 0.06 f
	+KM4	77.9 ± 1.37 e	0.39 ± 0.03 e	2.53 ± 0.14 d	1.37 ± 0.08 d	3.90 ± 0.13 d	0.25 ± 0.03 c

Values indicate means ± SE (*n* = 5). In the same column, values followed by similar letters are not significantly different.

**Table 4:** Levels of osmoprotectants and antioxidants in maize leaf in the presence or absence of *Serratia liquefaciens* KM4 under saline conditions.

NaCl (mM)	*Serratia liquefaciens* KM4	Proline (mg g^−1^ DW)	Soluble Sugars (mg g^−1^ DW)	Proteins (mg g^−1^ DW)	Total Free Amino Acids (mg g^−1^ DW)	Phenols (µmol g^−1^ FW)	Flavonoid (µmol g^−1^ FW)
0	−KM4	1.42 ± 0.17 f	27.54 ± 1.13 d	23.04 ±1.51 e	10.16 ± 0.13 f	7.51 ± 0.41 b	1.24 ± 0.14 b
	+KM4	1.77 ± 0.12 e	29.13 ± 1.47 a	23.93 ±1.25 d	11.03 ± 0.17 e	8.26 ± 0.33 a	1.51 ± 0.11 a
80	−KM4	2.25 ± 0.14 d	25.88 ± 1.38 e	24.81 ±1.28 c	12.14 ± 1.14 d	6.03 ± 0.27 e	1.05 ±0.16 d
	+KM4	2.91 ± 0.18 c	28.04 ± 1.29 c	26.42 ±1.13 b	15.26 ± 1.32 b	7.22 ± 0.35 c	1.17 ± 0.21 c
160	−KM4	3.41 ± 0.21 b	22.14 ± 1.37 f	25.77 ±1.18 b	14.17 ± 2.13 c	5.04 ± 0.29 f	0.98 ± 0.10 e
	+KM4	3.98 ± 0.19 a	28.89 ± 1.27 b	33.27 ±1.24 a	17.11 ± 2.24 a	6.11 ±0.42 d	1.07 ±0.12 d

Values indicate means ± SE (*n* = 5). In the same column, values followed by similar letters are not significantly different.

**Table 5:** Levels of H_2_O_2_, µmol g^−1^ FW), MDA (nmol g^−1^ FW), EL (%), and gas exchange parameters in maize leaves in the presence or absence of *Serratia liquefaciens* KM4 under salt stress.

NaCl (mM)	KM4	H_2_O_2_	MDA	EL (%)	*P_n_* (μmol m^2^ s^−1^)	*E* (mmol m^2^ s^−1^)	*g_s_* (mol m^2^ s^−1^)
0	−KM4	17.8 ± 1.11 e	6.9 ± 1.16 e	48.3 ± 2.15 e	13.24 ± 0.08 b	1.88 ± 0.05 b	0.09 ± 0.07 b
	+KM4	15.1 ± 1.34 f	5.1 ± 1.21 f	42.5 ± 2.03 f	14.91 ± 0.07 a	2.06 ± 0.11 a	0.12 ± 0.09 a
80	−KM4	19.8 ± 1.47 c	11.3 ± 1.24 c	69.2 ± 2.89 b	9.54 ± 0.05 d	1.54 ± 0.05 d	0.06 ±0.04 d
	+KM4	18.1 ±1.32 d	9.4 ± 1.72 d	55.7 ±2.13 d	10.98 ± 0.15 c	1.72 ± 0.08 c	0.07 ± 0.08 c
160	−KM4	22.4 ± 1.22 a	24.8 ± 1.27 a	77.4 ± 2.26 a	7.11 ± 0.13 f	1.17 ± 0.09 f	0.03 ± 0.05 f
	+KM4	20.1 ± 1.14 b	15.7 ± 1.82 b	66.8 ± 2.91 c	8.87 ± 0.15 e	1.33 ± 0.07 e	0.05 ± 0.01 e

Values indicate means ± SE (*n* = 5). In the same column, values followed by similar letters are not significantly different.
